# Defined metabolic states shape T cell fate and function across culture conditions

**DOI:** 10.3389/fimmu.2025.1703095

**Published:** 2025-11-06

**Authors:** Kayla Sylvester, Natasha Karassina, Anthony C. Lauer, Gediminas Vidugiris, Jolanta Vidugiriene

**Affiliations:** Research and Development, Promega Corporation, Madison, WI, United States

**Keywords:** T cell metabolism, ex vivo expansion, immunometabolism, bioluminescent assays, glycolysis, metabolic profiling, adoptive cell therapy, memory T cells

## Abstract

**Introduction:**

T cell metabolism is a key determinant of immune function and therapeutic efficacy, yet current expansion protocols often neglect how culture conditions influence metabolic programming. We employed a modular, low-input bioluminescent assay platform to profile how media, activation strength, and metabolic perturbation define metabolic trajectories that persist through early expansion and influence downstream outcomes.

**Methods:**

A multifactorial experimental design was used to evaluate early T-cell activation across media (ICXF, TexMACS, RPMI+FBS) and activators (TransAct, Dynabeads, ImmunoCult). Low-input bioluminescent assays were used to quantify metabolic cofactors (ATP, NAD^+^, NADP(H)), reducing capacity, and nutrient usage (glucose, lactate, malate). Conditions that yield metabolically distinct phenotypes were selected for deeper analysis of proliferation, cytokine secretion, cytotoxicity, and flow cytometric profiling. To validate and functionally confirm these phenotypes, pathway-specific metabolic inhibitors were introduced in follow-up experiments.

**Results:**

By measuring intracellular ATP, NAD^+^, NADP(H), reducing capacity, and nutrient flux, we identified media- and activation-specific metabolic states that emerged upon T-cell activation and persisted through early expansion. ICXF with TransAct promoted a glycolytic, NAD-rich phenotype associated with rapid expansion. In contrast, TexMACS with ImmunoCult supported oxidative metabolism, enriched for T_SCM_-like cells, and enhanced cytotoxicity despite slower growth. Early lactate levels strongly predicted downstream expansion (r = 0.68, p < 0.0001), highlighting glycolytic activity as a key determinant of proliferative potential. Functional validation with pathway-specific inhibitors revealed media-dependent vulnerabilities, highlighting distinct metabolic wiring.

**Conclusion:**

This approach enables predictive, multiplexed metabolic profiling using minimal sample input and offers a scalable strategy to optimize T-cell manufacturing for memory enrichment and cytotoxic potency.

## Introduction

Adoptive T-cell therapy has emerged as an innovative treatment strategy in cancer immunotherapy, particularly for hematological malignancies. Yet its broader clinical success is hindered by several challenges, including limited persistence of transferred cells, antigen escape, and the immunosuppressive tumor microenvironment (1–3). One critical and often underappreciated factor contributing to these limitations is the metabolic fitness of T cells, especially during ex vivo expansion, where intense stimulation can drive terminal differentiation and functional exhaustion (4–8). Efforts to improve T cell function increasingly focus on modulating their metabolic state, particularly during early activation when reprogramming first begins (9–12).

Upon activation, naïve T cells exit their quiescent, catabolic state and initiate profound metabolic reprogramming, marked by increased uptake of glucose and amino acids to support rapid growth and proliferation (13–15). This shift redirects metabolism from mitochondrial oxidative phosphorylation to an anabolic program dominated by aerobic glycolysis, fueling the biosynthetic demands of clonal expansion and effector differentiation (16–18).

In contrast, the formation and maintenance of memory T cells are sustained by a metabolically distinct state that favors mitochondrial oxidative metabolism, particularly enhanced fatty acid oxidation, supporting long-term survival and self-renewal (19, 20). These divergent metabolic programs are orchestrated by a combination of intrinsic factors, including transcriptional and epigenetic regulators, and extrinsic cues such as nutrient availability, cytokine signals (e.g., IL-2, IL-15), and the strength and duration of T cell receptor (TCR) stimulation (21–25).

Under ex vivo conditions, however, standard T-cell expansion protocols are designed to rapidly maximize cell yield and typically emphasize strong and sustained activation in nutrient-rich environments (26–28). These conditions preferentially promote effector-like differentiation with sustained glycolytic metabolism, yielding cell populations that differ metabolically and phenotypically from the heterogeneous subsets generated *in vivo* (29, 30). Consequently, ex vivo–expanded T cells often exhibit diminished persistence and functional capacity after *in vivo* transfer, underscoring the need to mimic physiological differentiation cues during manufacturing (31, 32).

Thus, assessing metabolic state during *in vitro* expansion provides a framework for optimizing culture conditions and improving the functional quality of cell therapy products. Strategies to enhance metabolic fitness have included cytokine modulation (e.g., IL-7 and IL-15), nutrient restriction or reprogramming, and the use of pharmacologic agents to promote mitochondrial metabolism and memory-like differentiation (33, 34). Memory-like CAR T cells, which exhibit enhanced oxidative metabolism, demonstrate superior persistence and antitumor activity, whereas glycolysis-driven CAR T cells are more prone to exhaustion and reduced efficacy (35–38).

Despite the growing recognition that metabolism shapes therapeutic outcomes, most manufacturing protocols still rely on phenotypic markers such as CD69/CD25 expression or cytokine secretion, which may not reliably reflect underlying metabolic fitness. Efforts to integrate metabolic assessments into manufacturing pipelines have been limited by the complexity and low throughput of existing technologies (39, 40). Techniques like Seahorse extracellular flux analysis and stable isotope tracing provide mechanistic depth but are not readily scalable for routine use (41).

In this study, to enhance metabolic T-cell fitness during ex vivo expansion, we employed a suite of sensitive, easy-to-use bioluminescent assays to profile key metabolic parameters under varying culture conditions (42, 43). By measuring intracellular ATP levels and redox cofactors (NAD(P)(H)), alongside extracellular metabolites such as glucose, lactate, and malate, we achieved sensitive, multiplexed analysis of energy status, redox balance, and nutrient utilization using minimal sample input. Integrating these metabolic profiles with functional readouts, we demonstrate that media composition and activation strength jointly shape early metabolic programs—particularly glycolytic activity and cofactor availability—which in turn influence proliferative capacity and differentiation into memory- or effector-like phenotypes. Together, these insights provide a foundation for rationally designing culture conditions that yield T-cell products with enhanced functionality and therapeutic potential.

## Materials and methods

### Donor material and T cell preparation

Peripheral blood mononuclear cells (PBMCs) were isolated from leukapheresis products (Leukopaks; STEMCELL Technologies, Cat. #70500) obtained from three healthy adult donors. Samples were collected under IRB-approved protocols provided by the vendor.

PBMCs were isolated by density gradient centrifugation at 300 × g for 10 minutes and resuspended in isolation buffer consisting of PBS with 1% BSA and 2 mM EDTA at a concentration of 5 × 10^7^ cells/mL. CD3^+^ T cells were enriched via negative selection using the EasySep Human T Cell Isolation Kit (STEMCELL Technologies, Cat. #17951) according to the manufacturer’s protocol.

Isolated T cells were cryopreserved in CryoStor CS10 (STEMCELL Technologies, Cat. #07930) using a controlled-rate freezer and stored in liquid nitrogen. Prior to use, cells were thawed and rested overnight in TexMACS medium (Miltenyi Biotec, Cat. #130-097-196) at 37°C and 5% CO_2_. Both non-activated and activated T cells were maintained under these conditions.

Cell counts and viability were assessed using ViaCount reagent on a Guava easyCyte cytometer (Cytek Biosciences) and were recorded throughout all experiments. These measurements were used to monitor cell health and to normalize metabolite measurements on a per-cell basis, as well as to calculate secretion rates and integrated cell-hours.

### Early T-cell activation and metabolic remodeling

T cells from 2–3 donors were activated at 1 × 10^6^ cells/mL in 6-well plates in either TexMACS or ImmunoCult-XF T cell Expansion medium (ICXF, STEMCELL Technologies, Cat. #10981). Cells were activated using either T cell TransAct (Miltenyi Biotec, Cat. #130-111-160) or ImmunoCult Human CD3/CD28 T Cell Activator (STEMCELL Technologies, Cat. #10971) and supplemented with IL-7 and IL-15 each at 2.5 ng/mL (Miltenyi Biotec, Cat. #130-095–361 and #130-095-764).

On Day 3, metabolic activity was evaluated using bioluminescent assays (see Bioluminescent Metabolite Assays). Flow cytometry was used to measure CD25 and CD69 expression (see Flow Cytometry), and cytokine secretion (IFN-γ and TNF-α) was quantified from Day 2 supernatants (see Cytokine Assays). Average cell volume was calculated based on mean cell diameter reported by the ViCell automated cell counter (Beckman Coulter).

### Metabolic profiling across activation conditions

A full-factorial design assessed three media (TexMACS, ICXF, RPMI 1640 [Gibco, Cat. #11875093] supplemented with 10% FBS [Gibco, Cat. #A5670401]), four activators (TransAct, ImmunoCult, Dynabeads Human T-Activator CD3/CD28 [Thermo Fisher Scientific, Cat. #11161D] at 1:1 and 2:1 bead-to-cell ratios), and two cytokine regimens: IL-2 at 30 IU/mL (IL2) or 60 IU/mL (IL2h), and IL-7/IL-15 each at 2.5 ng/mL (IL7/15) or 5 ng/mL (IL7/15h), yielding 48 unique conditions (**Table 1**). This screen was performed using T cells from a single donor to enable broad condition sampling. T cells were seeded and activated at 1 × 10^6^ cells/mL in 384-well plates (Corning, Cat. #3570) and harvested on Day 3.

Metabolic profiling included intracellular ATP, NAD^+^, NADP(H), reducing potential, and extracellular glucose, lactate, and malate levels (see Bioluminescent Metabolite Assays). These seven features were Z-score normalized prior to dimensionality reduction and clustering. Principal component analysis (PCA) was performed in Python using scikit-learn (v1.5.2) to reduce dimensionality. The top three principal components, which together explained approximately 68% of the total variance, were used for K-means clustering (k = 4), with cluster number determined by silhouette analysis. PCA visualizations were generated using matplotlib and seaborn, with samples color-coded by media or cluster.

### Validation across donors

Eight representative activation conditions (**Table 2**) were selected from the 48-condition screen to represent each of the four K-means–defined metabolic clusters. Selections were based on cluster membership, condition uniqueness, and variation across media, activator, and cytokine combinations to ensure phenotypic breadth and experimental diversity. Each of these conditions was subsequently tested in 2–3 independent donors to assess reproducibility.

T cells were seeded and activated at 1 × 10^6^ cells/mL in non-treated 24-well plates using media, activators, and cytokines shown in **Table 1**. On Day 3, metabolic profiling was conducted as described in Section 8, with lactate secretion and intracellular NADP(H) levels serving as the primary readouts. Flow cytometry was used to evaluate CD25 and CD69 expression (see Flow Cytometry), and cytokine secretion (IFN-γ and TNF-α) was measured from culture supernatants (see Cytokine Assays).

**Table 1:** Activation conditions overview.

Category	Details
Media	ICXF, RPMI+FBS, TexMACS
Activators	Dynabeads 1:1, Dynabeads 2:1, ImmunoCult, TransAct
Cytokines	IL2, IL2 h, IL7/15, IL7/15 h
Total Unique Conditions	48
Samples per Condition	4

### T cell expansion and metabolite analysis

Following activation and metabolic profiling on Day 3 (Validation Across Donors), T cells from representative conditions were seeded at 5 × 10^5^ cells per well into GREX24 vessels (Wilson Wolf Manufacturing, Cat. #80192M) for expansion. Activators were removed at the time of transfer, and cytokines were replenished and maintained throughout the culture period.

Supernatants were collected on Day 7 and used to measure glucose consumption, lactate secretion, and malate accumulation. Fold expansion was calculated relative to the number of viable cells seeded into GREX24 vessels on Day 3. Viability and expansion were evaluated on Day 7, with Day 10 included for supplemental comparison. Lactate:glucose and lactate:malate ratios were calculated from the secretion rates, and Pearson correlation analysis was used to assess the relationship between lactate accumulation on Day 3 and fold expansion through Day 7.

### Metabolic inhibition

To investigate condition-specific metabolic dependencies, T cells were cultured in either ICXF with ImmunoCult activator or TexMACS with TransAct activator, each supplemented with IL-7 and IL-15 (2.5 ng/mL each). At activation on Day 0, cells were treated with 5 mM 2-deoxyglucose (2DG; Sigma-Aldrich) to inhibit glycolysis, 20 µM Rotenone (Sigma-Aldrich) to inhibit mitochondrial complex I, or 0.2 µM Antimycin A (Sigma-Aldrich) to inhibit mitochondrial complex III. On Day 3, cells were transferred to GREX24 vessels with activators removed, and inhibitors and cytokines were replenished. Intracellular ATP and NAD were measured on Day 3. Lactate secretion and fold expansion were assessed from Day 3 to Day 7. All metrics were reported as percent inhibition relative to untreated, activated controls. Statistical comparisons were conducted using Welch’s unpaired *t*-test to account for unequal variances between groups.

### Post-expansion phenotype and function

To evaluate long-term effects, T cells from selected conditions were analyzed post-expansion. Memory phenotype was assessed on Day 10 by flow cytometry using CD45RA, CD62L, CD4, and CD8 antibodies (see Flow Cytometry). T cell subsets were gated as follows: T_SCM_ (stem cell memory, CD45RA^+^CD62L^+^CD95^+^), T_CM_ (central memory, CD45RA^-^CD62L^+^), T_EM_ (effector memory, CD45RA^-^CD62L^-^), and T_EMRA_ (terminal effector, CD45RA^+^CD62L^-^).

Cytotoxicity assays were performed using the HiBiT Ramos T-Cell Killing Bioassay (JA1411; Promega) according to the manufacturer’s CD8^+^ T Cell TDCC Assay Protocol. Briefly, T cells were co-cultured with Ramos target cells expressing HiBiT at defined effector-to-target (E:T) ratios of 1:0.3, 1:0.6, 1:1, 1:2.5, 1:5, 1:10, and 1:20, in the presence of 0.92 ng/mL blinatumomab. Specific lysis was calculated relative to spontaneous release (no effector cells) and maximum lysis (digitonin-treated targets). AUC was used to quantify overall cytotoxic activity across the E:T response curves. The assay was performed using T cells from two independent donors, each tested in technical triplicate.

### Bioluminescent metabolite assays

Bioluminescent metabolite assays were performed using Promega kits and read on a GloMAX Discover plate reader (Cat. #GM3000). Assays were set up according to the manufacturer’s protocols.

Intracellular ATP, NAD^+^, and NADP(H) were quantified using CellTiter-Glo 2.0 (Cat. #G9242), NAD/NADH-Glo (Cat. #G9071), and NADP/NADPH-Glo (Cat. #G9081), respectively. Reducing potential was measured using the RealTime-Glo MT Cell Viability Assay (Cat. #G9711). For kinetic readouts, RealTime-Glo luminescence was recorded hourly for 72 hours (equivalent to 3 days) using a SparkCyto plate reader (Tecan, Cat. #30085834) maintained at 37°C and 5% CO_2_.

Extracellular metabolites (glucose, lactate, malate) were measured from supernatants collected at the indicated timepoints after activation. Supernatants were acidified with 0.4 N HCl to halt enzymatic activity, neutralized with 0.5 M Trizma base, and stored at −20°C. Samples were diluted using an Echo Acoustic Liquid Handler (Beckman Coulter) and analyzed using Promega kits for glucose (Cat. #J6021), lactate (Cat. #J5021), and malate (Cat. #J8021).

Extracellular metabolite concentrations were determined by comparing sample values to media-only controls: for lactate and malate, background levels were subtracted, while glucose consumption was calculated by subtracting sample concentrations from the initial media-only baseline. All measurements were converted to concentrations using internal assay standards. Secretion and consumption rates were calculated based on time elapsed and integrated cell-hours, representing the cumulative viable cell burden over the measurement period.

### Flow cytometry

Flow cytometry was used to assess activation and memory markers. CD25 (BV605, BioLegend, Cat. #302636) and CD69 (Pacific Blue, BioLegend, Cat. #310932) were assessed on Day 3 (**Supplementary Figure S1**). CD4 (APC, Miltenyi, Cat. #130-113-210), CD8 (FITC, Miltenyi, Cat. #130-113-875), CD45RA (PE/Cy7, BioLegend, Cat. #304112), and CD62L (BV785, BioLegend, Cat. #304830) were assessed on Day 10. Samples were acquired using a Guava easyCyte 12HT cytometer (Cytek) and analyzed using FlowJo v10. Gating strategy included exclusion of debris and doublets followed by quadrant gating on CD45RA and CD62L within CD4^+^ or CD8^+^ populations (see **Supplementary Figure S4**).

### Cytokine assays

Cytokines (IL-2, IFN-γ, TNF-α) were measured from cell culture supernatants using luminescent immunoassay kits (Promega, Cat. #W6020, W6040, W6050). Assays were performed according to the manufacturer’s instructions using internal assay standards provided. Supernatants were diluted in PBS containing 0.1% BSA to ensure measured values fell within the linear range of the assay.

### Statistical analyses

**Figure 1:**
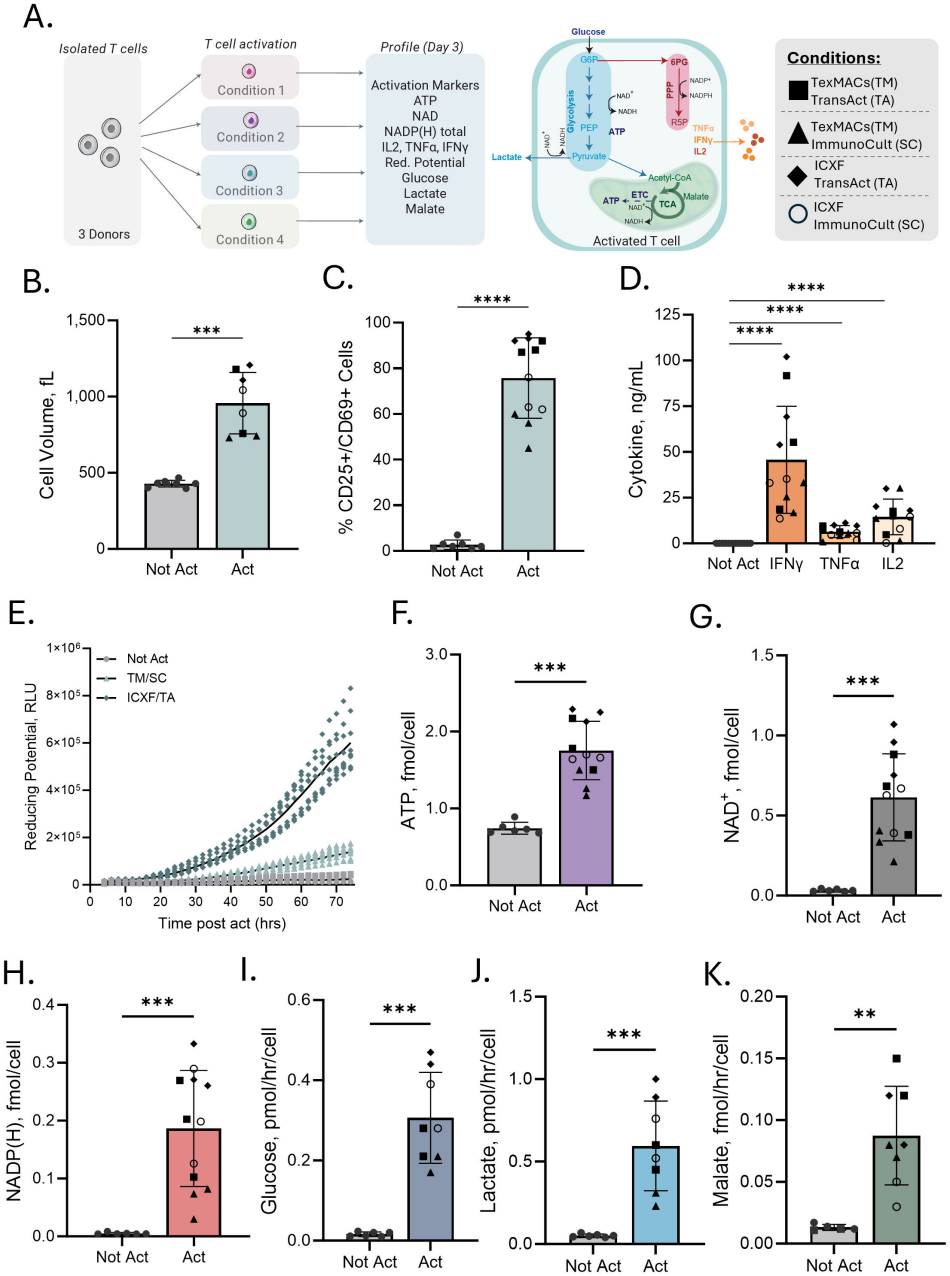
Multiparametric assessment identifies variation in T-cell activation across conditions. **(A)** Schematic of the experimental workflow and metabolic pathways assessed. CD3^+^ T cells from 2–3 donors were stimulated under four defined activation/media conditions (TexMACS [TM] or ICXF media combined with ImmunoCult [SC] or TransAct [TA] CD3/CD28 activators) and profiled on Day 3 for activation markers, cytokine secretion, and metabolic features. **(B–K)** Comparison of not activated (Not Act) and activated (Act) T cells. **(B)** Average cell volume (fL). **(C)** Percentage of CD25^+^ or CD69^+^ T cells measured by flow cytometry. **(D)** Cytokine secretion (IFNγ, TNFα, IL-2) measured 48 h post-activation. Not Act samples, which showed minimal or no cytokine production, are grouped together for display. Statistical comparisons were made between each Act condition and the Not Act group for each cytokine. **(E)** Metabolic reducing potential (measured as relative luminescence units, RLU) over 72 h for three conditions: Not Act, TM + SC, and ICXF + TA. **(F–H)** Intracellular ATP, NAD+, and total NADP + NADPH levels (all in fmol/cell). **(I, J)** Glucose consumption and lactate secretion (both in pmol/hr/cell) measured in culture media. **(K)** Malate accumulation levels (fmol/hr/cell). Each graph includes data from 2–3 donors. Shapes represent stimulation conditions. Each data point represents the average of 2–3 biological replicates (independent cultures) for a given donor and condition. Statistical comparisons used two-tailed, unpaired Mann–Whitney tests. Significance levels: p < 0.01 (**), p < 0.001 (***), p < 0.0001 (****).

All statistical analyses were performed using GraphPad Prism v10 (GraphPad Software) unless otherwise specified. Differences between two groups, such as activated vs. not activated T cells in **Figure 1**, were assessed using two-tailed, unpaired Mann–Whitney U tests, selected due to unequal variances, non-normal distributions, and the use of unpaired samples from independent cultures. For comparisons involving more than two groups, one-way ANOVA followed by Tukey’s multiple comparisons test was used to assess significance across conditions. For inhibitor studies where variances differed between media conditions, Welch’s unpaired t-test was employed to account for unequal variances and compare the magnitude of inhibition between matched media environments (e.g., TexMACS vs ICXF).

Principal component analysis (PCA) and K-means clustering were conducted using Python and the scikit-learn v1.5.2 library. Seven metabolic features, ATP, NAD^+^, total NADP(H), reducing potential, glucose consumption, lactate secretion, and malate levels, were Z-score normalized using StandardScaler to ensure all features contributed equally regardless of their dynamic range. The top three principal components, which explained approximately 68% of the total variance, were used for downstream clustering and visualization. K-means clustering (k = 4) was applied on these components, with cluster number determined via silhouette analysis. Visualization was performed using matplotlib v3.10.0 and seaborn v0.13.2.

Correlation analyses were conducted using Pearson’s correlation coefficient (r) to evaluate relationships between metabolic parameters (e.g., lactate secretion) and downstream outcomes (e.g., fold expansion). These analyses were used to identify predictive associations between early metabolic activity and subsequent functional performance.

Sample sizes, statistical tests, and replicate numbers are reported in the respective figure legends. Statistical test selection was guided by sample structure (paired vs. unpaired), variance equality, and distribution assumptions, each confirmed per analysis.

### Ethics statement

Human samples were obtained as leukopak products (Cat. #70500) under IRB-approved protocols via STEMCELL Technologies. Informed consent was obtained in accordance with the Declaration of Helsinki.

## Results

### Early T-cell activation is coupled to rapid metabolic remodeling

Early metabolic reprogramming shapes T cell fate, but tools to monitor these transitions during early activation remain limited. To address this, we stimulated T cells from three independent donors under four defined conditions combining two CD3/CD28-based activators (TransAct and ImmunoCult) with two commonly used media (ICXF and TexMACS). On Day 3 post-activation, cells were assessed using a panel of bioluminescent assays focused on metabolic activity, including energy production, redox balance, and nutrient consumption, alongside conventional activation markers (**Figure 1A**).

T cells displayed hallmark signs of early activation in all conditions, with increased cell size and elevated expression of CD25 and/or CD69 (**Figures 1B, C**, **Supplementary Figure S1**). Although all groups showed activation, cytokine secretion differed substantially. IL-2, IFN-γ, and TNF-α levels were elevated across conditions, but with distinct production patterns between them (**Figure 1D**), providing a functional context for interpreting the associated metabolic changes.

To monitor metabolic activity during early activation, we used the RealTime-Glo MT Viability assay, which reports NAD(P)H-dependent reductase activity over time in live cells (44). This approach captures dynamic changes in metabolic activity during the priming phase, when proliferation has not yet begun but metabolic engagement is increasing. All activated conditions showed higher luminescence than non-activated controls over the first 72 hours, with substantial variation in magnitude and kinetics depending on the activation condition (**Figure 1E**).

Activation also led to substantial changes in intracellular metabolic cofactors, which reflect key aspects of cellular energy status and redox balance. ATP levels increased two- to three-fold relative to resting cells, rising from approximately 0.6–0.8 fmol/cell to 1.2–2.3 fmol/cell (**Figure 1F**). NAD^+^ levels were also elevated across conditions, consistent with increased catabolic activity (**Figure 1G**). The largest shifts were observed in total NADP(H), which increased 6- to 66-fold (**Figure 1H**), suggesting strong engagement of biosynthetic and redox-supporting pathways, with variation across activation environments. These changes were reliably detected using only 1,000 to 5,000 cells per assay point, highlighting the sensitivity and scalability of the assay platform.

To gain insight into metabolic pathway activity, media supernatants were analyzed to assess extracellular metabolite dynamics during activation. Glucose consumption and lactate secretion increased across all stimulation conditions, reflecting elevated glycolytic activity typical of early effector programming (**Figures 1I, J**). Malate accumulation was also observed, indicating ongoing metabolite processing that may reflect increased mitochondrial activity or intermediate cycling through the TCA cycle (**Figure 1K**). These measurements reveal how nutrients are consumed, and byproducts are released during activation, offering a distinct perspective from intracellular assays and helping infer which pathways are engaged.

Together, these results show that bioluminescent assays can sensitively detect early metabolic changes following T-cell activation. This approach captures shared metabolic shifts associated with activation as well as condition-specific differences that emerge across diverse culture environments.

### Metabolic profiling reveals media-specific phenotypes

**Figure 2:**
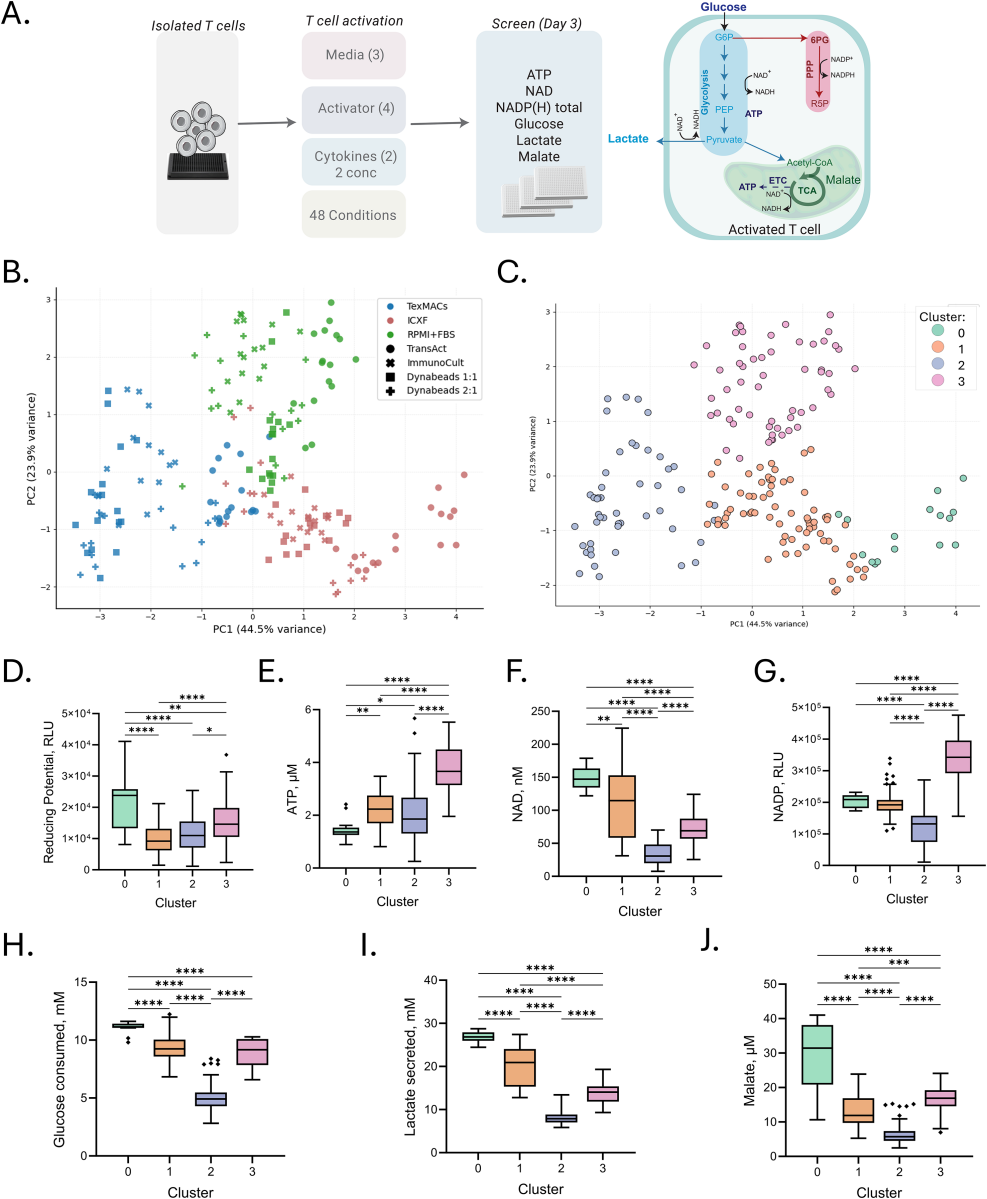
Activation medium drives metabolic profile of T cells. **(A)** Schematic of the experimental workflow and metabolic pathways investigated. T cells from a single donor were activated under 48 conditions combining three media types, four CD3/CD28-based activators, and two cytokine cocktails at two concentrations. Cells were harvested on Day 3 for metabolic profiling. **(B, C)** Principal component analysis (PCA) of intracellular metabolite profiles. **(B)** PCA colored by medium and marked by activator to visualize condition-dependent variation. **(C)** PCA colored by K-means cluster (k = 4), identifying four metabolically distinct groups. **(D–J)** Metabolite levels grouped by K-means cluster. **(D)** Metabolic reducing potential (measured as relative luminescence units, RLU). **(E–G)** Intracellular metabolite levels: ATP (µM), NAD+ (nM), and total NADP(H) (RLU). **(H, I)** Extracellular metabolite concentrations: glucose consumption and lactate secretion (both in mM). **(J)** Malate levels (µM). Each point represents the average of four technical replicates for a given activation condition. Box plots represent Tukey distribution: boxes span the interquartile range (IQR), whiskers extend to 1.5× IQR, and diamonds indicate outliers. Statistical comparisons were performed using one-way ANOVA followed by Tukey’s multiple comparisons test. Significance levels are indicated as follows: p < 0.05 (*), p < 0.01 (**), p < 0.001 (***), and p < 0.0001 (****).

Building on our initial findings of condition-dependent metabolic differences during early activation, we applied our assay suite as a screening tool for metabolic phenotyping of activated T cells. To capture global metabolic patterns, we employed a combinatorial experimental design (**Table 2**) and used principal component analysis (PCA) to dissect the influence of medium, activation method, and cytokine supplementation on the metabolic state of T cells (**Figure 2A**).

**Table 2:** Representative activation conditions used for donor validation.

Condition #	Media	Activator	Cytokine(s)
1	ICXF	ImmunoCult (SC)	IL7/15
2	ICXF	TransAct (TA)	IL7/15
3	ICXF	TransAct (TA)	IL2
4	TexMACS (TM)	Dynabeads (Dyna)	IL2
5	TexMACS (TM)	ImmunoCult (SC)	IL7/15
6	TexMACS (TM)	TransAct (TA)	IL7/15
7	RPMI+FBS	ImmunoCult (SC)	IL7/15
8	RPMI+FBS	TransAct (TA)	IL2

Each condition reflects a unique medium, activator, and cytokine combination selected from the 48-condition screen (**Figure 2**). Abbreviations in parentheses match those used in figure labels.

To reduce dimensionality and visualize trends, PCA was applied to seven key metabolic features: ATP, total NADP(H), NAD, reducing potential, lactate, glucose, and malate. The first two principal components explained 68% of the total variance. PC1 was primarily associated with glycolytic activity (glucose consumption, lactate secretion, NAD^+^ levels), while PC2 was driven by energy and redox status (ATP, total NADP(H)) (**Figure 2B**). Samples segregated distinctly by media, indicating that the culture environment is the dominant determinant of metabolic phenotype.

To formalize these trends, we applied K-means clustering to the standardized metabolic data. The optimal number of clusters (k = 4) was selected based on silhouette analysis, revealing four discrete metabolic states (**Figure 2C**). One-way ANOVA followed by Tukey HSD *post-hoc* tests confirmed highly significant differences (p < 0.001) in all metabolic markers across clusters (**Figures 2D–J**).

Cluster 0, composed exclusively of ICXF samples, exhibited the highest glucose, lactate, and malate levels but the lowest ATP levels, indicative of a highly glycolytic and potentially metabolically stressed state. Cluster 1, also ICXF-derived, displayed reduced glycolytic flux and elevated ATP and NAD^+^, suggesting a more energetically balanced, yet glycolysis-dependent phenotype. Cluster 2, dominated by TexMACS samples, had the lowest glucose uptake and lactate secretion while maintaining high ATP levels, consistent with a more oxidative and energy-efficient profile. Cluster 3, composed primarily of RPMI samples (with some TexMACS and ICXF), was characterized by the highest ATP and total NADP(H) levels and intermediate glycolytic activity, reflecting a metabolically balanced and biosynthetically active phenotype. The detailed composition of each cluster is provided in the supplemental material (**Supplementary Table S1**).

### Validation of metabolic and functional profiles across representative conditions

**Figure 3:**
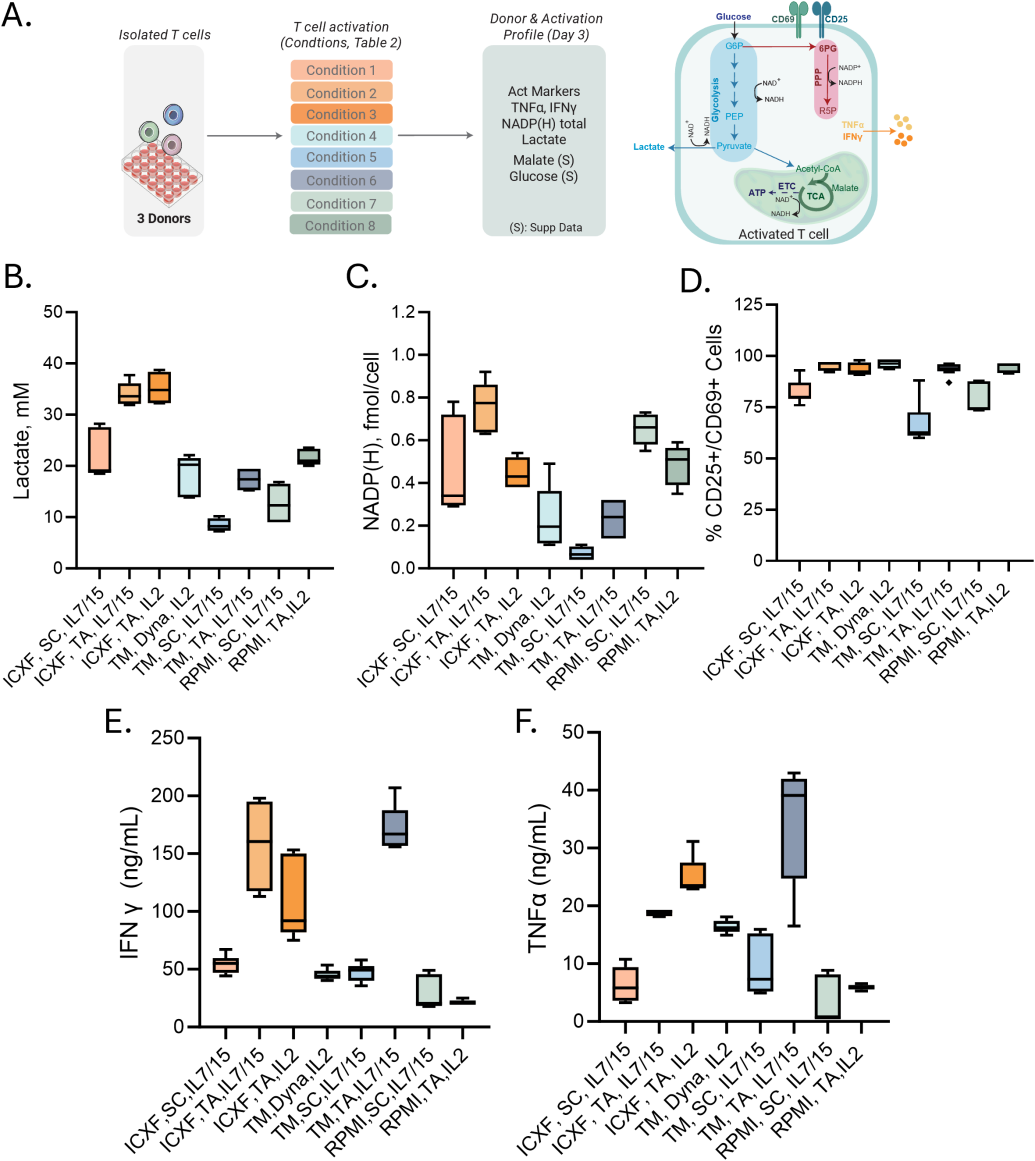
Metabolic and functional diversity across representative T-cell activation conditions. **(A)** Schematic of the validation workflow. Eight representative activation conditions were selected from the 48-condition screen (**Table 1**; abbreviations as defined there). Cells were harvested on Day 3 for metabolic and functional profiling. **(B–F)** Summary of Day 3 measurements. **(B)** Lactate secretion (mM) measured from culture media. **(C)** Intracellular total NADP **(H)** levels (fmol/cell). **(D)** Percentage of CD25^+^ or CD69^+^ T cells measured by flow cytometry. **(E–F)** Cytokine secretion: IFNγ (ng/mL) **(E)** and TNFα (ng/mL) **(F)**. Data summarize two to three donors tested in 2–3 experiments each. Box plots represent Tukey distribution. Shapes represent different donors. Statistical comparisons were performed using one-way ANOVA followed by Tukey’s multiple comparisons test. Most features showed statistically significant differences between conditions. Exact values are reported in **Supplementary Table S2**.

To determine whether the metabolic phenotypes observed in our initial screen were consistent across donors and independent experiments, we selected eight representative activation–medium combinations spanning the four K-means–defined metabolic clusters (**Table 1**, **Figure 3A**). These conditions were chosen to capture the diversity of metabolic profiles observed in the screen and included variation in media, activators, and cytokine conditions to ensure broad phenotypic representation.

For this analysis, we focused on lactate and NAD(P)H, two features that contributed most to the first two principal components in the PCA shown in **Figure 2B, C**. Lactate reflects glycolytic activity associated with PC1, while NAD(P)H serves as a marker of anabolic redox status represented by PC2. As shown in **Figures 3B, C**, these metabolic profiles were consistently reproduced across multiple donors and experimental replicates. T cells activated in ICXF (clusters 0 and 1) exhibited higher lactate secretion and elevated NAD(P)H levels compared to those cultured in TexMACS or RPMI (clusters 2 and 3). These results demonstrate that early metabolic profiles are shaped predominantly by culture medium, with overall patterns remaining stable across donor backgrounds.

However, when comparing responses within the same medium, differences between activators also became apparent. TransAct stimulation resulted in higher lactate secretion and increased NAD(P)H levels relative to ImmunoCult activator (SC), with Dynabeads (Dyna) producing intermediate values (**Figure 3C**). These trends suggest that stronger activation enhances both glycolytic and anabolic metabolism. This is further supported by flow cytometric analysis (**Figure 3D**), where conditions associated with greater metabolic activity also exhibited higher expression of CD25 and CD69, consistent with more robust TCR signaling.

To explore the relationship between early metabolic activity and functional output, we measured cytokine secretion across the same set of conditions. In this context, the strength of stimulation appeared to be the primary factor influencing effector cytokine production. TransAct consistently induced higher levels of IFN-γ and TNF-α compared to ImmunoCult, across all media. Cytokine levels were generally lower in RPMI-supported cultures, regardless of activator used.

In summary, these findings demonstrate that early metabolic states are shaped primarily by culture medium and are consistent across donors and experiments. Importantly, these adaptations are established within the first 72 hours following activation, before detectable proliferation, during a critical window when cells initiate the biosynthetic and energetic programs required for downstream expansion.

### Expansion dynamics are coupled to media-specific metabolism

**Figure 4:**
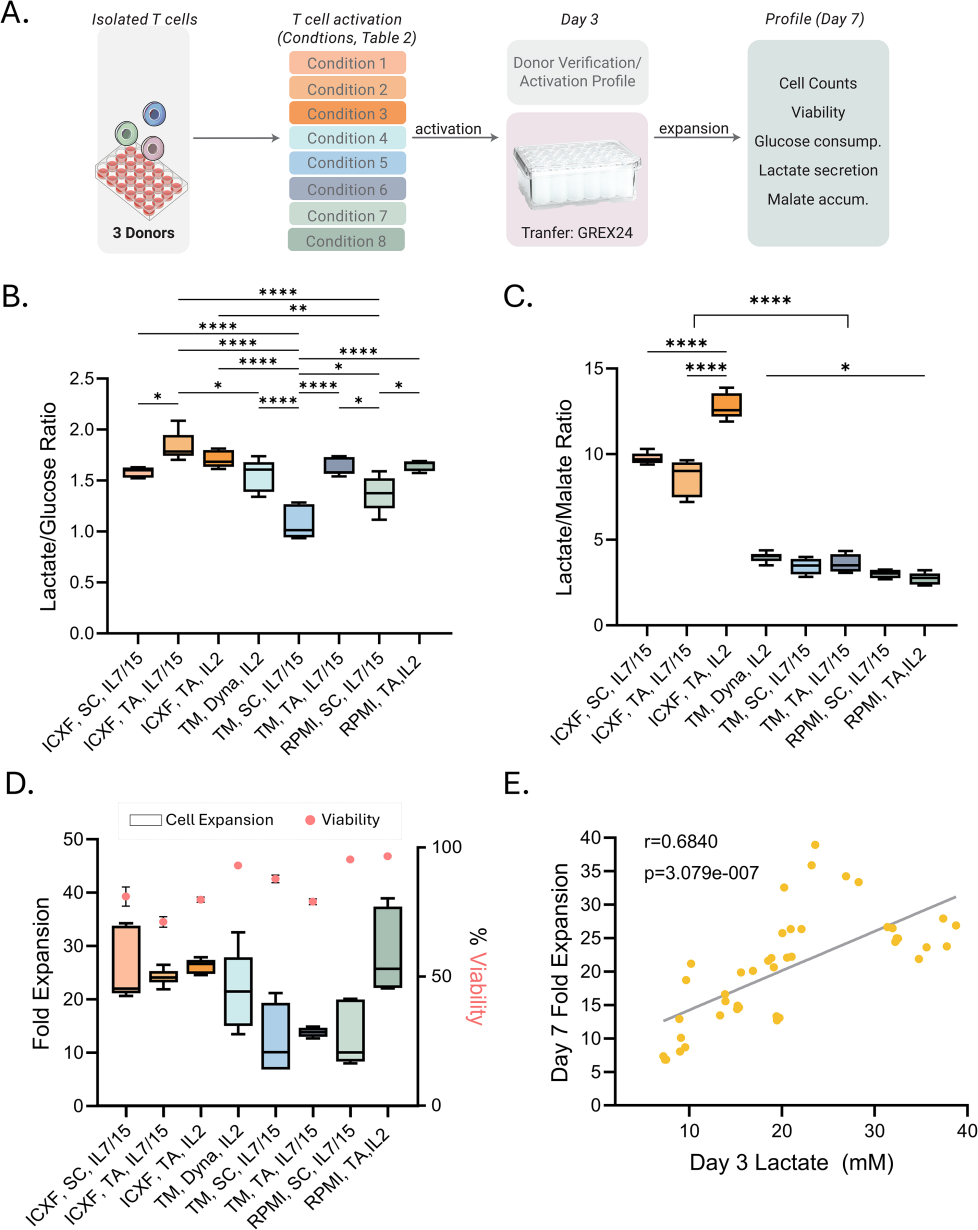
Metabolic signatures predict expansion outcomes following activation. **(A)** Schematic of the expansion workflow. T cells from 2–3 donors were activated under eight representative conditions (**Table 1**; abbreviations as defined there) and transferred to GREX24 vessels on Day 3 for continued culture. **(B, C)** Metabolic ratios calculated from the rate of metabolite secretion or consumption between Day 3 and Day 7: lactate-to-glucose ratio **(B)** and lactate-to-malate ratio **(C)**. **(D)** Fold expansion from Day 3 to Day 7 is shown as box plots (left axis). Viability on Day 7 is overlaid as pink dots (right axis). **(E)** Correlation between lactate levels on Day 3 (mM) and fold expansion through Day 7. Pearson correlation: r = 0.6840, p = 3.079e–07. Box plots **(B–D)** show Tukey distribution. Statistical comparisons were performed for panels B–C using one-way ANOVA followed by Tukey’s multiple comparisons test. For panel C, all pairwise comparisons were performed, but significance is summarized using a single bracket to indicate the overall difference between TexMACS and ICXF conditions for clarity. Significance levels are indicated as follows: p < 0.05 (*), p < 0.01 (**), and p < 0.0001 (****).

To determine whether media-dependent metabolic phenotypes observed during activation were sustained during expansion and whether they correlated with cell proliferation, we assessed both metabolic activity and growth between Day 3 and Day 7 post activation (**Figure 4A**).

Glucose consumption, lactate production, and malate accumulation were measured to profile metabolic activity (**Supplementary Figure S3**). The patterns established during activation were largely maintained during early stages of expansion, with the highest metabolic activity observed in ICXF medium. To facilitate comparisons, we calculated two metabolic ratios: lactate-to-glucose (**Figure 4B**), reflecting glycolytic efficiency, and lactate-to-malate (**Figure 4C**), providing a relative measure of glycolysis versus mitochondrial metabolism. Similar approaches have been used to infer metabolic preferences in T cell subsets, with high lactate-to-glucose ratios indicating glycolytic skewing typical of effector T cells, and lower ratios suggesting oxidative metabolism characteristic of memory subsets (45, 46). While these ratios do not quantify flux directly, they offer a practical overview of dominant metabolic outputs.

As shown in **Figure 4**, T cells activated in ICXF, particularly with TransAct and IL-2, exhibited consistently higher lactate-to-glucose and lactate-to-malate ratios, indicating sustained glycolytic activity. In contrast, lower lactate-to-malate ratios in TexMACS and RPMI suggested greater mitochondrial involvement.

These metabolic differences aligned with total cell expansion (**Figure 4D**) and proliferation rates. T cells in ICXF expanded more rapidly, with the shortest doubling times (19–22 hours), compared to slower expansion in TexMACS (19–34.5 hours) and RPMI (18–32 hours). Across conditions, higher glycolytic activity was associated with greater fold expansion, linking early metabolic programming to proliferative capacity.

Importantly, lactate levels at Day 3 correlated strongly with total expansion by Day 7 (Pearson r = 0.68, p < 0.0001; **Figure 4E**), suggesting early glycolytic activity predicts short-term expansion. This relationship was not maintained between Day 7 and Day 10, when proliferation declined (**Supplementary Figure S3D**), likely due to nutrient depletion, increased cell density, or a metabolic transition toward a less proliferative state.

### Targeted inhibition confirms media-specific metabolic dependencies

To validate the distinct metabolic profiles established under each culture condition, we assessed their responses to glycolytic and mitochondrial inhibition. Cells were cultured in the presence of 2-deoxyglucose (2DG; a glycolysis inhibitor), Rotenone (a Complex I inhibitor), or Antimycin A (a Complex III inhibitor), and metabolic and proliferative metrics were assessed.

**Figure 5:**
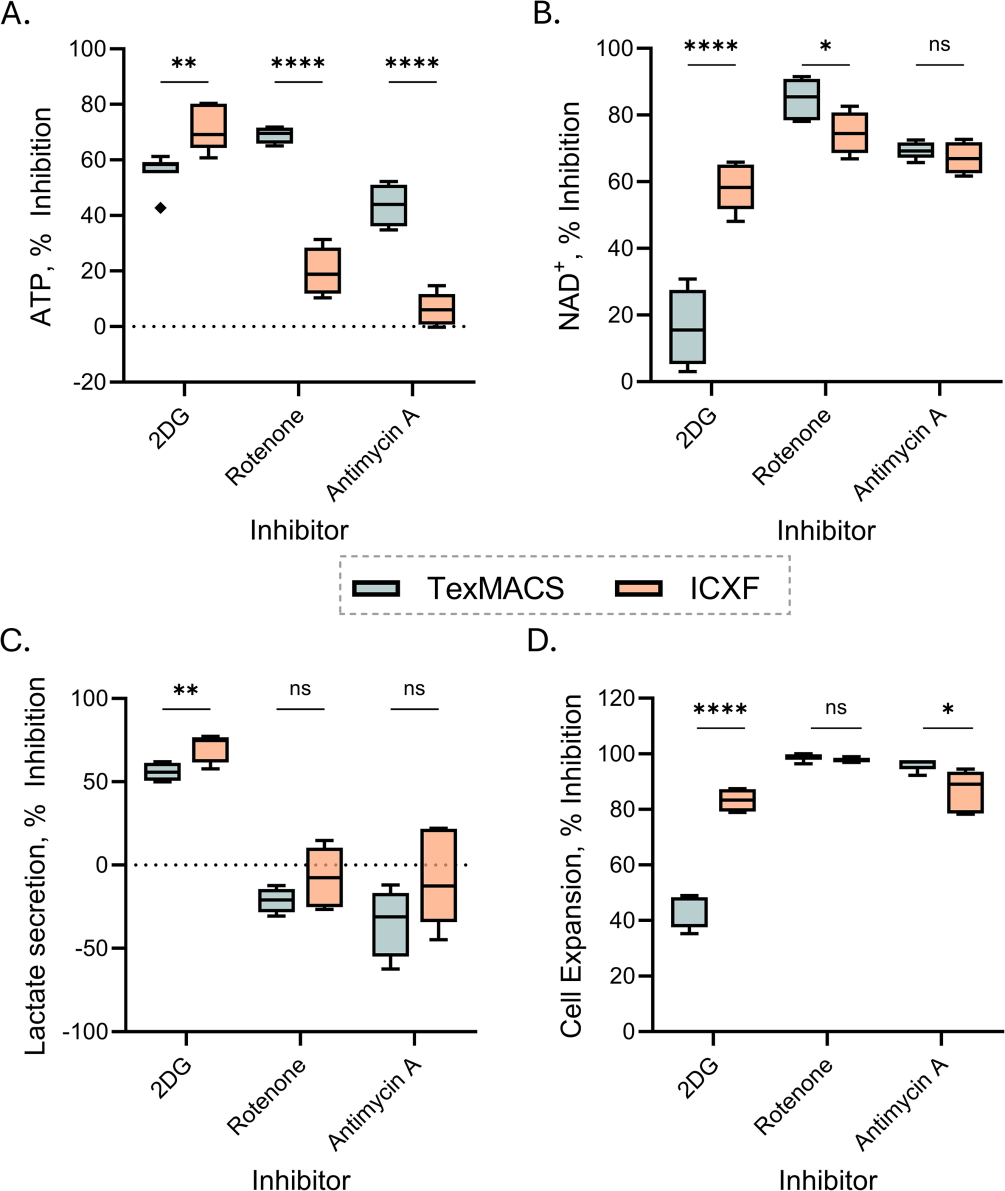
Targeted inhibition confirms media-dependent metabolic differences in T cells. **(A–D)** Percent inhibition of metabolic and expansion metrics following treatment with 2DG, Antimycin A, or Rotenone, relative to untreated controls in matched conditions. Data are shown for TexMACS (blue) and ImmunoCult-XF (orange) media. **(A, B)** Intracellular ATP **(A)** and NAD **(B)** levels measured on Day 3. **(C)** Percent inhibition of lactate secretion rate from Day 3 to Day 7. **(D)** Growth rate inhibition calculated from fold expansion between Day 3 and Day 7. Box plots represent Tukey distribution. Data summarize two donors. Statistical comparisons were performed using Welch’s unpaired t-tests. Significance levels are indicated as follows: ns=not significant, p < 0.05 (*), p < 0.01 (**), and p < 0.0001 (****).

Inhibition of glycolysis with 2DG significantly reduced intracellular ATP levels in both media conditions. ICXF-expanded cells exhibited greater inhibition (~60–80%) than those in TexMACS (~43–61%) (**Figure 5A**). NAD levels were also more strongly inhibited in ICXF, with more than 2-fold greater inhibition compared to TexMACS (**Figure 5B**). These reductions in energy metabolites corresponded with marked inhibition of both lactate secretion and cell expansion in ICXF (**Figures 5C, D**), whereas TexMACS-expanded cells showed less inhibition of lactate secretion and a more modest reduction in proliferation. These data support a greater glycolytic dependence in ICXF and enhanced metabolic flexibility in TexMACS.

To assess mitochondrial contributions, T cells were treated with Rotenone or Antimycin A. Both inhibitors caused greater ATP inhibition in TexMACS (~69% for Rotenone and ~44% for Antimycin A) than in ICXF (~22% and ~4%, respectively) (**Figure 5A**), suggesting higher mitochondrial contribution to ATP production in TexMACS-expanded cells. NAD levels were more inhibited by Rotenone in TexMACS, while Antimycin A caused similarly high inhibition in both media without a significant difference (**Figure 5B**). Lactate secretion increased in response to mitochondrial inhibition, particularly in TexMACS-expanded cells, though the magnitude of this effect varied across donors (**Figure 5C**). This trend supports a compensatory shift toward glycolysis when mitochondrial respiration is impaired. Growth inhibition from Day 3 to Day 7 was observed in both media. Rotenone significantly reduced proliferation in both conditions, while Antimycin A caused greater inhibition in TexMACS, though substantial effects were also observed in ICXF (**Figure 5D**). These findings confirm that ICXF-expanded cells are more glycolysis-dependent, while TexMACS-expanded cells are more sensitive to mitochondrial inhibition, consistent with their distinct metabolic phenotypes. To determine whether these metabolic states translate into functional differences, we next assessed T cell phenotype and effector function.

### Metabolism environment directs T cell phenotype and function

Metabolic cues during early T-cell activation not only support effector functions but also play a pivotal role in shaping memory differentiation. T_SCM_ (stem cell memory) cells, in particular, are highly valued for their longevity and antitumor potency in adoptive therapies, and their generation has been linked to oxidative metabolic programming (35, 47, 48).

**Figure 6:**
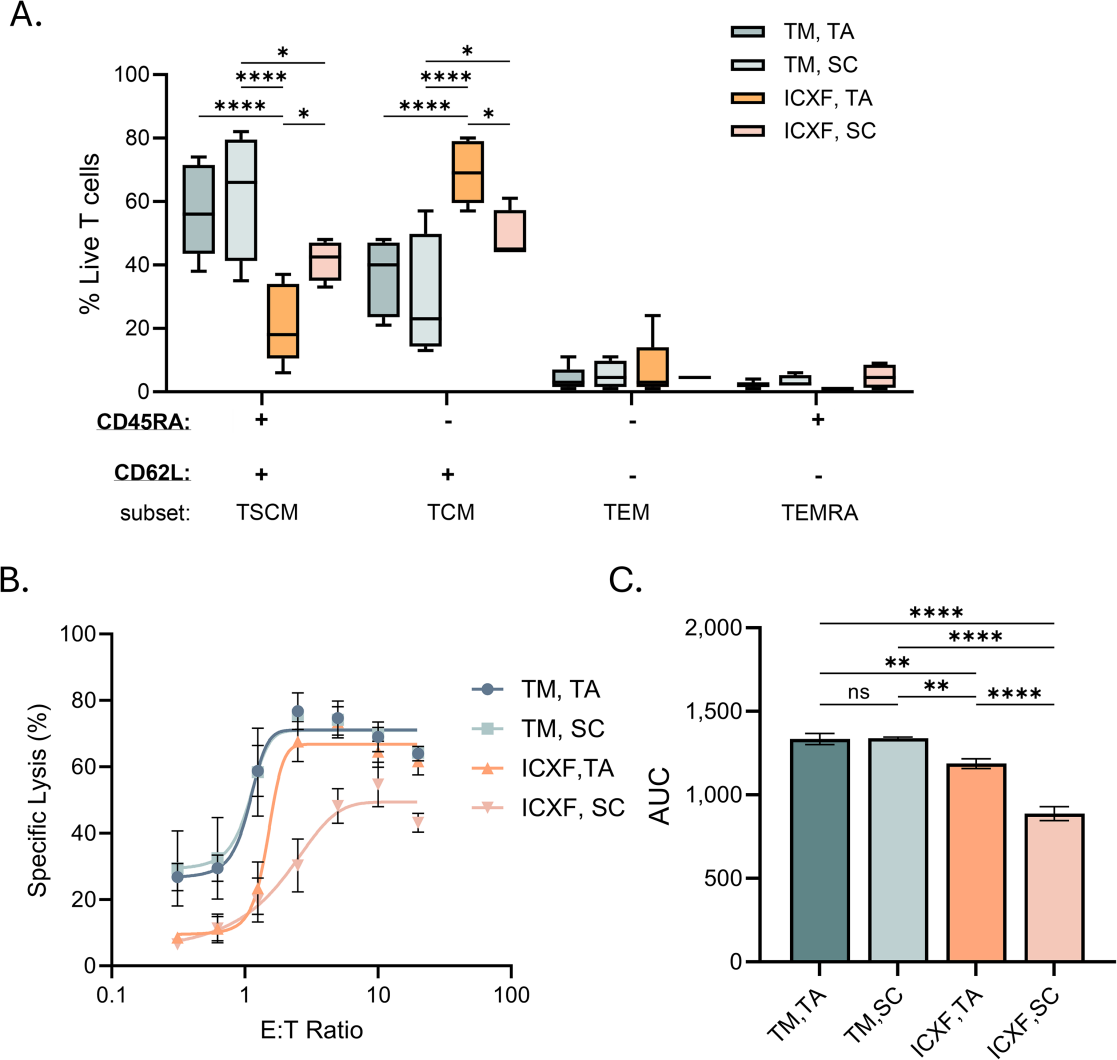
Functional responses of T cells activated in different media conditions. **(A–C)** Functional assessments were performed on Day 10 post-activation using T cells expanded under representative media and activation conditions. **(A)** Memory phenotype distribution of CD4^+^ and CD8^+^ T cell subsets assessed by flow cytometry using CD45RA and CD62L expression. Box plots represent Tukey distribution. **(B)** Specific lysis (%) of HiBiT Ramos target cells co-cultured with T cells and blinatumomab at seven effector-to-target (E:T) ratios. Lysis was calculated relative to spontaneous release (Ramos cells alone, no T cells) and maximum lysis (digitonin-treated Ramos). **(C)** Quantification of cytotoxic activity based on area under the curve (AUC) from E:T response curves. Data summarize two donors. Statistical comparisons were performed using one-way ANOVA with Tukey’s multiple comparisons test. Significance levels are indicated as follows: ns=not significant, p < 0.05 (*), p < 0.01 (**), and p < 0.0001 (****). TM, TexMACS; TA, TransAct; SC, StemCell ImmunoCult.

Given the distinct metabolic programs supported by ICXF and TexMACS during activation and expansion, we next examined whether these conditions influenced memory phenotype and effector function (**Supplementary Figure S4**). Phenotypic analysis revealed that T cells expanded in ICXF contained a higher proportion of CD45RA^-^CD62L^+^ cells, consistent with a T_CM_-like (central memory) phenotype. In contrast, TexMACS-expanded cells were enriched for CD45RA^+^CD62L^+^ cells, indicative of a T_SCM_-like population (**Figure 6A**). The development of T_SCM_ cells has been linked to mitochondrial respiration and reduced reliance on glycolysis, consistent with the more oxidative metabolic profile observed in TexMACS cultures.

Functionally, TexMACS-expanded T cells exhibited superior cytolytic activity against target cells compared to those expanded in ICXF (**Figures 6B, C**). This enhanced effector function aligns with the phenotypic and metabolic characteristics of T_SCM_ cells, which combine high proliferative potential with strong mitochondrial fitness.

These findings suggest that the metabolic environment during early activation not only determines expansion kinetics but also influences memory differentiation. By supporting a more oxidative metabolic program, TexMACS favors the generation of metabolically resilient and functionally potent T_SCM_-like cells, which may offer advantages in therapeutic applications.

## Discussion

Recent advances have highlighted that metabolic programming is not merely a byproduct of T-cell activation but a primary determinant of fate, function, and therapeutic efficacy (49–51). Our study supports and expands on this paradigm by showing that early metabolic profiling of T cells under defined culture conditions can predict proliferation, differentiation trajectories, and cytotoxic potential—key attributes of effective adoptive cell therapies.

While prior studies have profiled T cell metabolism using technologies such as Seahorse assays or transcriptomics, these approaches are often limited to end-point measurements or low throughput. In contrast, our study applies bioluminescent assays that enable repeated or real-time metabolic profiling across clinically relevant expansion conditions. This platform offers predictive insights using minimal cell input and straightforward, plate-based assays, making it readily implementable in both research and manufacturing settings without specialized equipment or complex workflows. Consistent with previous studies demonstrating the importance of glycolysis in supporting T-cell activation, biomass accumulation, and clonal expansion (52, 53), our data show that glycolytic activity is rapidly upregulated upon activation and correlates with early proliferative capacity. Culturing activated T cells in ICXF medium, which promotes strong glycolytic engagement, resulted in the highest proliferative rates, particularly when combined with potent activation via TransAct. This is in line with prior findings demonstrating that high glycolytic flux sustains mTORC1 activity and effector differentiation (53). However, such metabolic wiring also correlated with more differentiated T_CM_ and T_EM_ (effector memory) subsets, suggesting that excessive glycolysis may drive commitment at the expense of long-term persistence (7, 37).

By contrast, TexMACS medium fostered a more balanced metabolic profile, with moderate glycolysis and higher malate output, consistent with greater mitochondrial engagement. This balance correlated with an enrichment of T_SCM_ subsets, especially under milder activation with the ImmunoCult activator. This mirrors work by Gubser et al. (47) and Sukumar et al. (35), which showed that mitochondrial metabolism and spare respiratory capacity are hallmarks of T cells with greater *in vivo* persistence and antitumor efficacy. Notably, TexMACS-expanded cells also exhibited the strongest cytolytic function in our killing assays, underscoring the functional relevance of balanced metabolic states.

These metabolic profiles emerged within the first 72 hours, prior to significant proliferation, suggesting that metabolic priming during early activation sets a trajectory for downstream outcomes. This echoes recent reports that early nutrient sensing and redox balance influence epigenetic programming and transcriptional commitment (29, 48). Our use of bioluminescent metabolic assays allowed for high-sensitivity measurements using minimal input, offering a scalable, real-time window into these early decisions.

Importantly, while our findings highlight how defined metabolic environments guide T-cell outcomes *in vitro*, recent studies, and our own observations, underscore that T cells expanded ex vivo often diverge metabolically from their *in vivo* counterparts. A deeper understanding of these differences may inform the design of next-generation culture systems that more closely recapitulate the metabolic programs of physiologically activated T cells, ultimately enhancing the functionality and persistence of therapeutic products.

In conclusion, our results reinforce the concept that metabolism is a central regulator of T-cell fate. Future efforts to integrate metabolic assessment into manufacturing workflows may enable more precise engineering of T-cell products tailored to clinical goals, whether rapid tumor clearance or long-term immune surveillance.

## Data Availability

The original contributions presented in the study are included in the article/**Supplementary Material**. Further inquiries can be directed to the corresponding authors.

## References

[1] Brudno JN Maus MV Hinrichs CS Cells CART . and T-cell therapies for cancer: A translational science review. JAMA. (2024) 332:1924–35. doi: 10.1001/jama.2024.19462, PMID: 39495525 PMC11808657

[2] Dias J Garcia J Agliardi G Roddie C . CAR-T cell manufacturing landscape-Lessons from the past decade and considerations for early clinical development. Mol Ther Methods Clin Dev. (2024) 32:101250. doi: 10.1016/j.omtm.2024.101250, PMID: 38737799 PMC11088187

[3] Albelda SM . CAR T cell therapy for patients with solid tumours: key lessons to learn and unlearn. Nat Rev Clin Oncol. (2024) 21:47–66. doi: 10.1038/s41571-023-00832-4, PMID: 37904019

[4] Zebley CC Zehn D Gottschalk S Chi H . T cell dysfunction and therapeutic intervention in cancer. Nat Immunol. (2024) 25:1344–54. doi: 10.1038/s41590-024-01896-9, PMID: 39025962 PMC11616736

[5] Bacigalupa ZA Landis MD Rathmell JC . Nutrient inputs and social metabolic control of T cell fate. Cell Metab. (2024) 36:10–20. doi: 10.1016/j.cmet.2023.12.009, PMID: 38118440 PMC10872404

[6] Peng JJ Wang L Li Z Ku CL Ho PC . Metabolic challenges and interventions in CAR T cell therapy. Sci Immunol. (2023) 8:eabq3016. doi: 10.1126/sciimmunol.abq3016, PMID: 37058548

[7] Van der Vreken A Vanderkerken K De Bruyne E De Veirman K Breckpot K Menu E . Fueling CARs: metabolic strategies to enhance CAR T-cell therapy. Exp Hematol Oncol. (2024) 13:66. doi: 10.1186/s40164-024-00535-1, PMID: 38987856 PMC11238373

[8] Sudarsanam H Buhmann R Henschler R . Influence of culture conditions on ex vivo expansion of T lymphocytes and their function for therapy: current insights and open questions. Front Bioeng Biotechnol. (2022) 10:886637. doi: 10.3389/fbioe.2022.886637, PMID: 35845425 PMC9277485

[9] Shen L Xiao Y Zhang C Li S Teng X Cui L . Metabolic reprogramming by ex vivo glutamine inhibition endows CAR-T cells with less-differentiated phenotype and persistent antitumor activity. Cancer Lett. (2022) 538:215710. doi: 10.1016/j.canlet.2022.215710, PMID: 35489446

[10] Le Bourgeois T Strauss L Aksoylar HI Daneshmandi S Seth P Patsoukis N . Targeting T cell metabolism for improvement of cancer immunotherapy. Front Oncol. (2018) 8:237. doi: 10.3389/fonc.2018.00237, PMID: 30123774 PMC6085483

[11] Ramamurthy A Tommasi A Saha K . Advances in manufacturing chimeric antigen receptor immune cell therapies. Semin Immunopathol. (2024) 46:12. doi: 10.1007/s00281-024-01019-4, PMID: 39150566 PMC12054169

[12] Ye L Park JJ Peng L Yang Q Chow RD Dong MB . A genome-scale gain-of-function CRISPR screen in CD8 T cells identifies proline metabolism as a means to enhance CAR-T therapy. Cell Metab. (2022) 34:595–614.e14. doi: 10.1016/j.cmet.2022.02.009, PMID: 35276062 PMC8986623

[13] Geltink RIK Kyle RL Pearce EL . Unraveling the complex interplay between T cell metabolism and function. Annu Rev Immunol. (2018) 36:461–88. doi: 10.1146/annurev-immunol-042617-053019, PMID: 29677474 PMC6323527

[14] Van der Windt GJ Pearce EL . Metabolic switching and fuel choice during T-cell differentiation and memory development. Immunol Rev. (2012) 249:27–42. doi: 10.1111/j.1600-065X.2012.01150, PMID: 22889213 PMC3645891

[15] Klein Geltink RI Edwards-Hicks J Apostolova P O’Sullivan D Sanin DE Patterson AE . Metabolic conditioning of CD8+ effector T cells for adoptive cell therapy. Nat Metab. (2020) 2:703–16. doi: 10.1038/s42255-020-0256-z, PMID: 32747793 PMC10863625

[16] DePeaux K Delgoffe GM . Metabolic barriers to cancer immunotherapy. Nat Rev Immunol. (2021) 12:785–97. doi: 10.1038/s41577-021-00541-y, PMID: 33927375 PMC8553800

[17] Ma S Ming Y Wu J Cui G . Cellular metabolism regulates the differentiation and function of T-cell subsets. Cell Mol Immunol. (2024) 21:419–35. doi: 10.1038/s41423-024-01148-8, PMID: 38565887 PMC11061161

[18] O’Sullivan D . The metabolic spectrum of memory T cells. Immunol Cell Biol. (2019) 9:636–46. doi: 10.1111/imcb.12274, PMID: 31127964

[19] Pearce EL Walsh MC Cejas PJ Harms GM Shen H Wang LS . Enhancing CD8 T-cell memory by modulating fatty acid metabolism. Nature. (2009) 460:103–7. doi: 10.1038/nature08097, PMID: 19494812 PMC2803086

[20] Biasco L Izotova N Rivat C Ghorashian S Richardson R Guvenel A . Clonal expansion of T memory stem cells determines early anti-leukemic responses and long-term CAR T cell persistence in patients. Nat Cancer. (2021) 6:629–42. doi: 10.1038/s43018-021-00207-7, PMID: 34345830 PMC7611448

[21] Hunt EG Hurst KE Riesenberg BP Kennedy AS Gandy EJ Andrews AM . Acetyl-CoA carboxylase obstructs CD8+ T cell lipid utilization in the tumor microenvironment. Cell Metab. (2024) 36:969–983.e10. doi: 10.1016/j.cmet.2024.02.009, PMID: 38490211 PMC12010431

[22] Angelin A Gil-de-Gómez L Dahiya S Jiao J Guo L Levine MH . Foxp3 reprograms T cell metabolism to function in low-glucose, high-lactate environments. Cell Metab. (2017) 25:1282–1293.e7. doi: 10.1016/j.cmet.2016.12.018, PMID: 28416194 PMC5462872

[23] Si X Shao M Teng X Huang Y Meng Y Wu L . Mitochondrial isocitrate dehydrogenase impedes CAR T cell function by restraining antioxidant metabolism and histone acetylation. Cell Metab. (2024) 36:176–192.e10. doi: 10.1016/j.cmet.2023.12.010, PMID: 38171332

[24] Toledano Zur R Atar O Barliya T Hoogi S Abramovich I Gottlieb E . Genetically engineering glycolysis in T cells increases their antitumor function. J Immunother Cancer. (2024) 12:e008434. doi: 10.1136/jitc-2023-008434, PMID: 38964783 PMC11227835

[25] Zhou J Jin L Wang F Zhang Y Liu B Zhao T . Chimeric antigen receptor T (CAR-T) cells expanded with IL-7/IL-15 mediate superior antitumor effects. Protein Cell. (2019) 10:764–9. doi: 10.1007/s13238-019-0643-y, PMID: 31250350 PMC6776495

[26] Ma S Dahabieh MS Mann TH Zhao S McDonald B Song W-S . Nutrient-driven histone code determines exhausted CD8+ T cell fates. Science. (2024) 387:1–15. doi: 10.1126/science.adj3020, PMID: 39666821 PMC11881194

[27] MacPherson S Keyes S Kilgour MK Smazynski J Chan V Sudderth J . Clinically relevant T cell expansion media activate distinct metabolic programs uncoupled from cellular function. Mol Ther Methods Clin Dev. (2022) 24:380–93. doi: 10.1016/j.omtm.2022.02.004, PMID: 35284590 PMC8897702

[28] Leney-Greene MA Boddapati AK Su HC Cantor JR Lenardo MJ . Human plasma-like medium improves T lymphocyte activation. iScience. (2020) 23:100759. doi: 10.1016/j.isci.2019.100759, PMID: 31887663 PMC6941860

[29] Ma EH Verway MJ Johnson RM Roy DG Steadman M Hayes S . Metabolic profiling using stable isotope tracing reveals distinct patterns of glucose utilization by physiologically activated CD8+ T cells. Immunity. (2019) 51:856–870.e5. doi: 10.1016/j.immuni.2019.09.003, PMID: 31747582

[30] Frisch AT Wang Y Xie B Yang A Ford BR Joshi S . Redirecting glucose flux during *in vitro* expansion generates epigenetically and metabolically superior T cells for cancer immunotherapy. Cell Metab. (2025) 37:870–885.e8. doi: 10.1016/j.cmet.2024.12.007, PMID: 39879981 PMC12101091

[31] Adu-Berchie K Liu Y Zhang DKY Freedman BR Brockman JM Vining KH . Generation of functionally distinct T-cell populations by altering the viscoelasticity of their extracellular matrix. Nat BioMed Eng. (2023) 7:1374–91. doi: 10.1038/s41551-023-01052-y, PMID: 37365267 PMC10749992

[32] Sin WX Jagannathan NS Teo DBL Kairi F Fong SY Tan JHL . A high-density microfluidic bioreactor for the automated manufacturing of CAR T cells. Nat BioMed Eng. (2024) 12:1571–91. doi: 10.1038/s41551-024-01219-1, PMID: 38834752

[33] Zhu M Han Y Gu T Wang R Si X Kong D . Class I HDAC inhibitors enhance antitumor efficacy and persistence of CAR-T cells by activation of the Wnt pathway. Cell Rep. (2024) 43:114065. doi: 10.1016/j.celrep.2024.114065, PMID: 38578828

[34] Nava Lauson CB Tiberti S Corsetto PA Conte F Tyagi P Machwirth M . Linoleic acid potentiates CD8+ T cell metabolic fitness and antitumor immunity. Cell Metab. (2023) 35:633–650.e9. doi: 10.1016/j.cmet.2023.02.013, PMID: 36898381

[35] Sukumar M Liu J Ji Y Subramanian M Crompton JG Yu Z . Inhibiting glycolytic metabolism enhances CD8+ T cell memory and antitumor function. J Clin Invest. (2013) 123:4479–88. doi: 10.1172/JCI69589, PMID: 24091329 PMC3784544

[36] Gattinoni L Lugli E Ji Y Pos Z Paulos CM Quigley MF . A human memory T cell subset with stem cell-like properties. Nat Med. (2011) 17:1290–7. doi: 10.1038/nm.2446, PMID: 21926977 PMC3192229

[37] Cappabianca D Pham D Forsberg MH Bugel M Tommasi A Lauer A . Metabolic priming of GD2 TRAC-CAR T cells during manufacturing promotes memory phenotypes while enhancing persistence. Mol Ther Methods Clin Dev. (2024) 32:101249. doi: 10.1016/j.omtm.2024.101249, PMID: 38699288 PMC11063605

[38] Pham DL Cappabianca D Forsberg MH Weaver C Mueller KP Tommasi A . Label free metabolic imaging to enhance the efficacy of Chimeric Antigen Receptor T cell therapy Nat. Biomed Eng. (2025). doi: 10.1038/s41551-025-01504-7, PMID: 40958004 PMC12445593

[39] Richelle A David B Demaegd D Dewerchin M Kinet R Morreale A . Towards a widespread adoption of metabolic modeling tools in biopharmaceutical industry: a process systems biology engineering perspective. NPJ Syst Biol Appl. (2020) 6:6. doi: 10.1038/s41540-020-0127-y, PMID: 32170148 PMC7070029

[40] Cadinanos-Garai A Flugel CL Cheung A Jiang E Vaissié A Abou-El-Enein M . High-dimensional temporal mapping of CAR T cells reveals phenotypic and functional remodeling during manufacturing. Mol Ther. (2025) 33:2291–309. doi: 10.1016/j.ymthe.2025.04.006, PMID: 40315840 PMC12126796

[41] Jang C Chen L Rabinowitz JD . Metabolomics and isotope tracing. Cell. (2018) 173:822–37. doi: 10.1016/j.cell.2018.03.055, PMID: 29727671 PMC6034115

[42] Leippe D Sobol M Vidugiris G Cali JJ Vidugiriene J . Bioluminescent assays for glucose and glutamine metabolism: high-throughput screening for changes in extracellular and intracellular metabolites. SLAS Discov. (2017) 22:366–77. doi: 10.1177/1087057116675612, PMID: 27803177

[43] Vidugiriene J Leippe D Sobol M Vidugiris G Zhou W Meisenheimer P . Bioluminescent cell-based NAD(P)/NAD(P)H assays for rapid dinucleotide measurement and inhibitor screening. Assay Drug Dev Technol. (2014) 12:514–26. doi: 10.1089/adt.2014.605, PMID: 25506801 PMC4270152

[44] Duellman SJ Zhou W Meisenheimer P Vidugiris G Cali JJ Gautam P . Bioluminescent, nonlytic, real-time cell viability assay and use in inhibitor screening. Assay Drug Dev Technol. (2015) 13:456–65. doi: 10.1089/adt.2015.669, PMID: 26383544 PMC4605357

[45] van der Windt GJW O’Sullivan D Everts B Huang SC Buck MD Curtis JD . CD8 memory T cells have a bioenergetic advantage that underlies their rapid recall ability. Proc Natl Acad Sci U.S.A. (2013) 110:14336–41. doi: 10.1073/pnas.1221740110, PMID: 23940348 PMC3761631

[46] O’Sullivan D van der Windt GJW Huang SC Curtis JD Chang CH Buck MD . Memory CD8^+^ T cells use cell-intrinsic lipolysis to support the metabolic programming necessary for development. Immunity. (2014) 41:75–88. doi: 10.1016/j.immuni.2014.06.005, PMID: 25001241 PMC4120664

[47] Gubser PM Bantug GR Razik L Fischer M Dimeloe S Hoenger G . Rapid effector function of memory CD8+ T cells requires an immediate-early glycolytic switch. Nat Immunol. (2013) 14:1064–72. doi: 10.1038/ni.2687, PMID: 23955661

[48] Ron-Harel N Santos D Ghergurovich JM Sage PT Reddy A Lovitch SB . Mitochondrial biogenesis and proteome remodeling promote one-carbon metabolism for T cell activation. Cell Metab. (2016) 24:104–17. doi: 10.1016/j.cmet.2016.06.007, PMID: 27411012 PMC5330619

[49] Dugnani E Pasquale V Bordignon C Canu A Piemonti L Monti P . Integrating T cell metabolism in cancer immunotherapy. Cancer Lett. (2017) 411:12–8. doi: 10.1016/j.canlet.2017.09.039, PMID: 28974448

[50] Claiborne MD . Manipulation of metabolic pathways to promote stem-like and memory T cell phenotypes for immunotherapy. Front Immunol. (2023) 13:1061411. doi: 10.3389/fimmu.2022.1061411, PMID: 36741362 PMC9889361

[51] Piscopo NJ Mueller KP Das A Hematti P Murphy WL Palecek SP . Bioengineering solutions for manufacturing challenges in CAR T cells. Biotechnol J. (2018) 13:10.1002/biot.201700095. doi: 10.1002/biot.201700095, PMID: 28840981 PMC5796845

[52] Buck MD O’Sullivan D Klein Geltink RI Curtis JD Chang CH Sanin DE . Mitochondrial dynamics controls T cell fate through metabolic programming. Cell. (2016) 166:63–76. doi: 10.1016/j.cell.2016.05.035, PMID: 27293185 PMC4974356

[53] Chang CH Curtis JD Maggi LB Jr Faubert B Villarino AV O’Sullivan D . Posttranscriptional control of T cell effector function by aerobic glycolysis. Cell. (2013) 153:1239–51. doi: 10.1016/j.cell.2013.05.016, PMID: 23746840 PMC3804311

