# Supplementary material for: Defined metabolic states shape T cell fate and function across culture conditions
Source: Front Immunol. 2025 Nov 6;16:1703095. doi: 10.3389/fimmu.2025.1703095 (PMC12631338; doi:10.3389/fimmu.2025.1703095)
Supplement: Supplementary file 1 [file Image1.pdf]

Supplemental Material

Table S1. Assignment of activation conditions to K-means clusters from Figure 2. Each row corresponds to one of the 48 conditions from the full-factorial metabolic screen, with associated media, activator, cytokine, and K-means cluster identity (Cluster 0–3) based on PCA of intracellular metabolite profiles.

Table S2. Statistical comparisons for Figure 3. One-way ANOVA with Tukey's multiple comparisons test was used to assess differences across the eight representative activation conditions. Reported p-values include comparisons for CD25, CD69, lactate secretion, total NADP(H) levels, and secretion of IFNγ and TNFα.

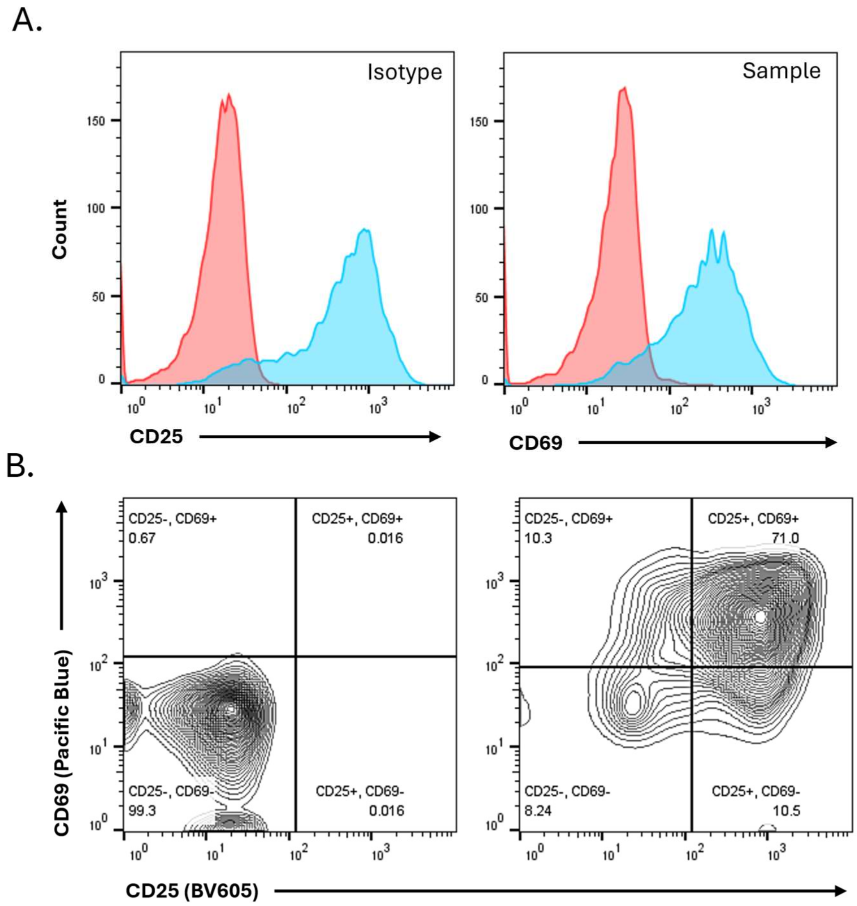

Figure S1. Gating strategy for CD25 and CD69. (A) Representative overlay of fluorescence intensity for isotype controls and an activated sample stained for CD25 and CD69. (B) Quadrant gating applied to nonactivated (left) and activated (right) T cells to identify CD25 and CD69 single- and dual-positive populations.

A.

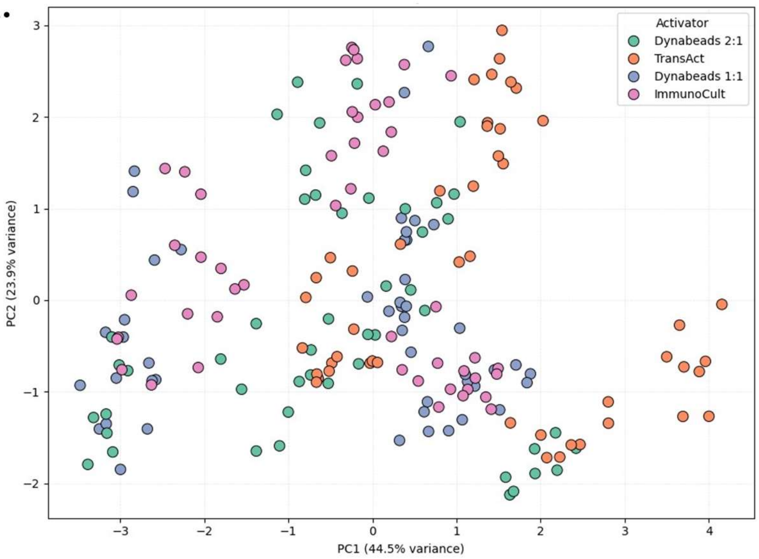

B.

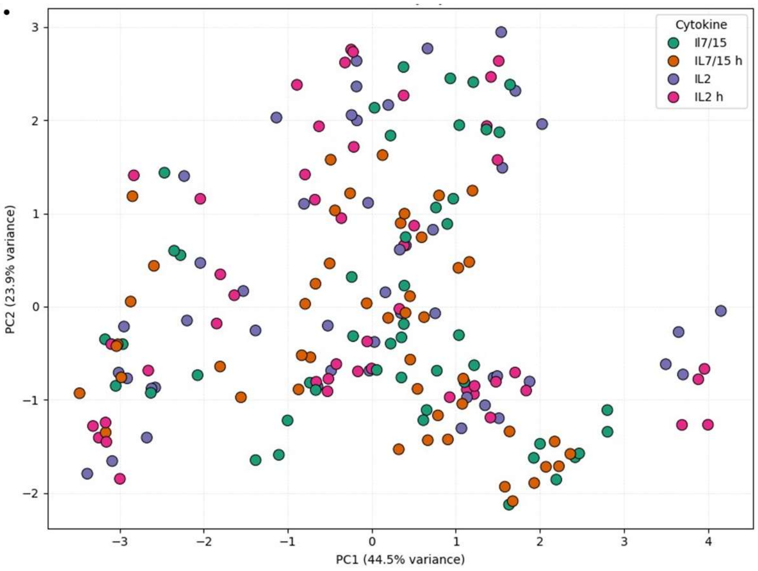

Figure S2. Principal component analysis colored by activator or cytokine. Principal component analysis of intracellular metabolite profiles from 48 activation conditions in a single donor. (A) PCA colored by CD3/CD28-based activator. (B) PCA colored by cytokine supplementation.

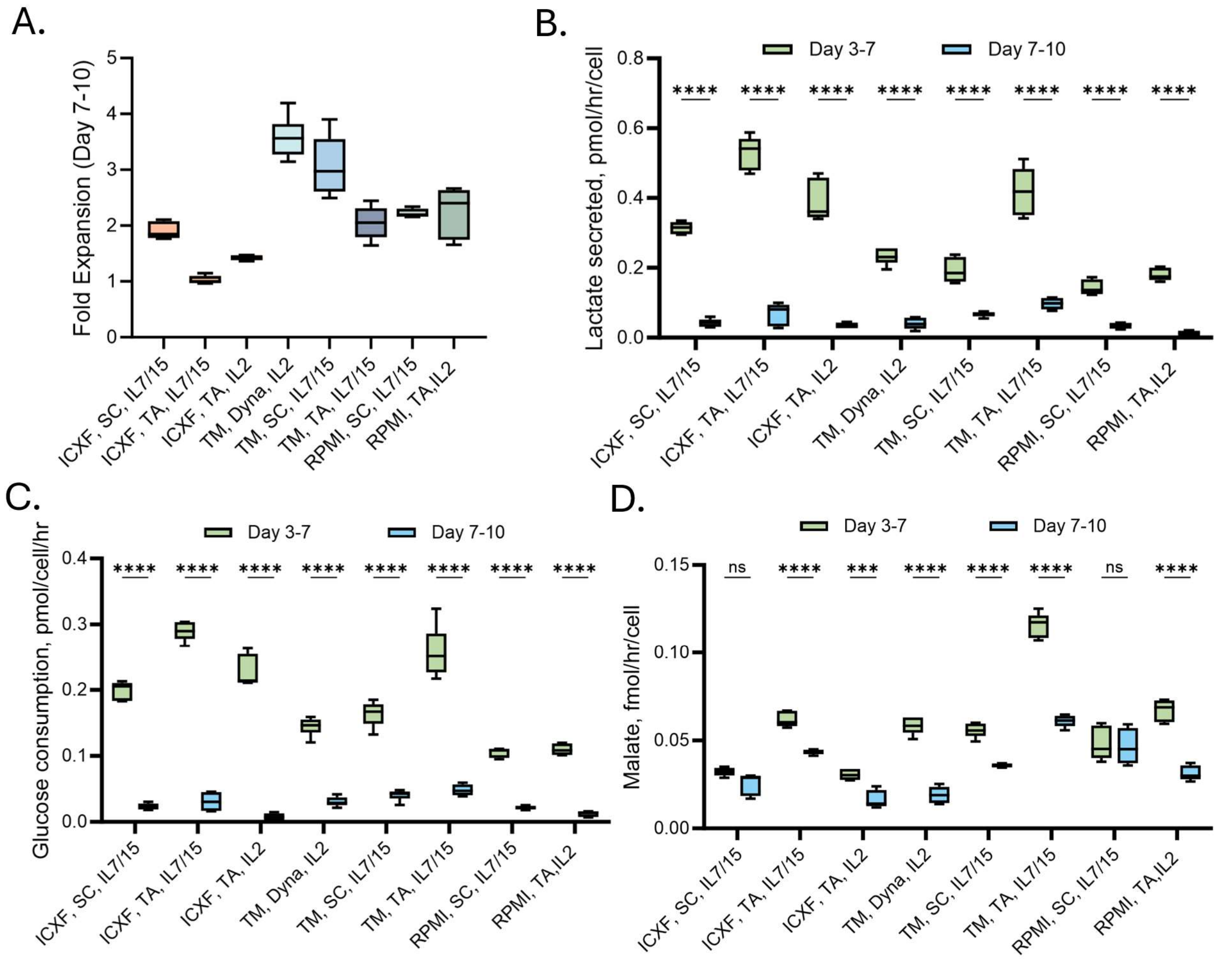

Figure S3. Fold expansion and metabolic activity from Day 7 to Day 10. (A) Fold expansion between Day 7 and Day 10 for each activation condition. (B–D) Metabolite secretion or consumption rates measured from Day 3–7 and Day 7–10, including (B) lactate secretion, (C) glucose consumption, and (D) malate accumulation. Data were collected from 2 donors, each tested in 2–3 independent biological replicates per condition. Statistical comparisons between time intervals were performed using two-way ANOVA with Tukey's multiple comparisons test. Box plots represent Tukey distribution.

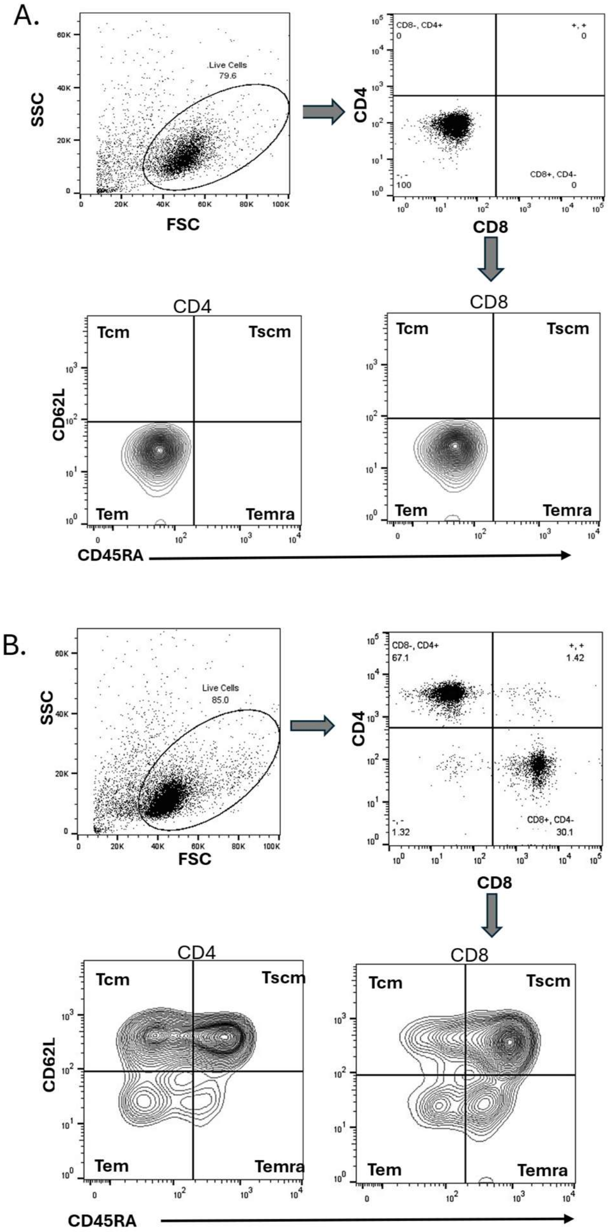

Figure S4. Gating strategy for memory T cell subset identification. Live cells were gated based on FSC and SSC, followed by separation into CD4⁺ and CD8⁺ T cell populations. Memory subsets were defined by CD45RA and CD62L expression: TSCM (CD45RA^{+}CD62L^{+}), TCM (CD45RA^{-}CD62L^{+}), TEM (CD45RA^{-}CD62L^{-}), and TEMRA (CD45RA^{+}CD62L^{-}). (A) Representative isotype control. (B) Representative sample showing CD4^{+} and CD8^{+} T cell subset distributions.
